# Effects of digital health education intervention on stress, anxiety and depression of patients with polycystic ovarian syndrome: study protocol for a single blinded randomized controlled trial

**DOI:** 10.3934/publichealth.2026007

**Published:** 2026-01-09

**Authors:** Sadia Akter, Farkhanda Mahjebin, Mohammad Delwer Hossain Hawlader, Md Moshiur Rahman, Sanmei Chen, Saori Kashima, Sheuly Akter, Shamit Dasgupta, Yoko Shimpuku

**Affiliations:** 1 Graduate School of Innovation and Practice for Smart Society, Hiroshima University, Japan; 2 Khwaja Yunus Ali Medical College and Hospital, Bangladesh; 3 Department of Public Health, North South University, Bangladesh; 4 NSU Global Health Institute (NGHI), North South University, Dhaka 1229, Bangladesh; 5 Graduate School of Biomedical and Health Sciences, Hiroshima University, Japan; 6 Center for the Planetary Health and Innovation Science, The IDEC Institute, Hiroshima University, Japan; 7 Environmental Health Sciences Laboratory, Graduate School of Advanced Science and Engineering, Hiroshima University, Japan; 8 Ministry of Health, Government of British Columbia, Canada

**Keywords:** polycystic ovarian syndrome, depression, anxiety, stress, mHealth, digital health education

## Abstract

**Background:**

Polycystic ovarian syndrome (PCOS) is an endocrine disorder that is connected to a range of gynecological symptoms. The disorder has both physical and mental health outcomes. While the physical symptoms are often expressed and diagnosed, the mental health outcomes are ignored and undiagnosed. To the best of our knowledge, in Bangladesh, although women are affected by the disease, research exploring the impact of digital health interventions on the mental health burden is scarce. Emerging evidence suggests that smartphone-based health education interventions could be helpful in alleviating the psychological burden associated with PCOS by improving awareness and self-management among patients.

**Objective:**

The aim of the study is to explore the impact of digital health education on the stress, anxiety, and depression levels among PCOS patients in Bangladesh.

**Methods:**

A single blinded, randomized controlled trial was being conducted at the Enayetpur of Sirajganj district at Khwaja Yunus Ali Medical College and Hospital. A total of 212 participants were randomly assigned to either the intervention group or the control group in the study. The intervention group received PCOS related health education via a mobile application reinforced with app notifications and bi-monthly phone calls from the researcher. Baseline Data collection was completed in April 2025. Endline data collection started in November 2025. The intervention was a six-month intervention that ran from May to October. The primary outcome, depression, stress and anxiety was measured using the DASS21 questionnaire. The secondary outcomes were serum hormone levels (cortisol and testosterone), body mass index (BMI), and knowledge about PCOS.

**Results:**

The baseline data collection was completed in April 2025. A total of 212 patient provided their consent to participate in the study. The participants were divided into groups of 106 individuals for the control and intervention groups.

**Trial registration:**

Japan Registry of Clinical Trials (jRCT2062240073), https://jrct.mhlw.go.jp/en-latest-detail/jRCT2062240073 and North South University Institutional Review Board approved this study (#2024/0RNSU/IRBII 002).

## 1. Introduction

Polycystic ovarian syndrome (PCOS) is an endocrine disorder with reproductive outcomes that has a global prevalence of 5%–20% among women [1],[2]. Patients who have PCOS suffer from irregular menstruation, acne, excessive body and facial hair, infertility, metabolic disorder, diabetes, heart disease and emotional instability. All these symptoms cause stress in patients, which subsequently leads to depression and anxiety [3]. Moreover, stress leads to a rise in cortisol level, which adversely affects the metabolism and cognitive functions [4],[5]. Additionally, the levels of testosterone and prolactin hormones are often increased considered to be closely related to the condition of PCOS. Increased testosterone causes hirsutism, acne, and male pattern hair loss, while increased prolactin causes decreased ovulation [6],[7].

Among these symptoms, infertility has a strong impact on the quality of life of the patients. Decreased ovulation and increased testosterone are related to infertility of patients with PCOS [8]. Infertility itself comes with immense stress, both psychologically and socially. Having PCOS along with infertility is a double disease burden that affects a woman's overall physical and mental health [9]. Although patients receive PCOS related symptomatic treatments from gynecologists, lifestyle modifications and behavior changes are equally important for the patients [10].

Currently, treatments of PCOS are limited to symptom specific treatments. The main treatment options are lifestyle modification, diet, and weight management [11],[12]. Albeit many harmful health impacts, the disease is being ignored in terms of awareness among the patients who are suffering every day [13]. This leads to compromised health conditions and immense psychological adverse outcomes, which lead to a poor quality of life [14].

In Bangladesh, women and girls of reproductive age with the condition are unaware of the disease and are left undiagnosed [15]. Women and girls receive medical treatments solely based on medications but not on lifestyle modifications [16]. Even though the body and mind are not separate entity, when it comes to patients' health, physical health tends to receive attention and priority, whereas mental health is mostly left undiagnosed or even unnoticed [17].

With the advancement of technology, many healthcare facilities and healthcare options are accessible to people. A diverse range of social media platforms provides an abundance of information. However, misinformation is not uncommon [17]–[19]. It is important to receive information that is structured, scientifically proven, and professionally approved [20]. This study aims to elucidate the effectiveness of digital health information on a patient's health, both physical and mental. The combined approach of lifestyle modification and traditional medical treatments may also help health professionals to utilize digital technologies to manage and educate patients.

The secondary outcome of this study, serum cortisol and testosterone levels, are commonly associated with psychological stress, which is the primary outcome of this study [21]. While studies showed that cortisol promotes abdominal fat accumulation and slows down weight loss, elevated testosterone contributes to male pattern baldness, hirsutism, and adversely affects a woman's appearance [22],[23]. Body image concerns play a vital role in increased psychological distress [24].

We hypothesize that health education through a smartphone-based application intervention will significantly reduce the levels of stress, anxiety, and depression among PCOS patients compared to usual treatments.

Additionally, our study hypothesizes that digital health education interventions will encourage lifestyle related behaviors that may reduce the testosterone and cortisol levels and eventually improve the depression, anxiety, and stress related to PCOS.

## 2. Materials and methods

### 2.1. Design

**Figure 1.**
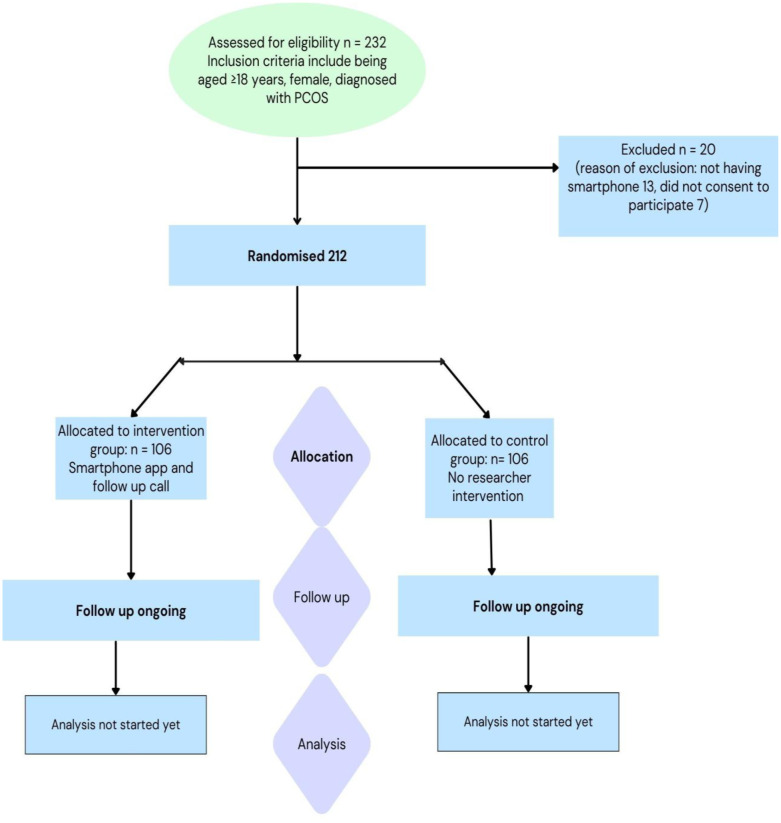
Participant recruitment flowchart.

This is a hospital-based intervention study that is prospective in nature and has two arms (1:1). This is a single blinded, single centered study where the participants were divided into either an intervention group or a control group. The participants were recruited from a rural private hospital of Bangladesh. The randomized controlled trial design strictly adheres to Consolidated Standards of Reporting Trial (CONSORT) [25] guidelines along with the Standard Protocol Items: Recommendations for Interventional Trial (SPIRIT) [26] guidelines. A schematic overview is shown in Figure 1.

### 2.2. Study setting and eligibility criteria

The study participants were selected from Enayetpur, which is under the administrative and demographic surveillance jurisdiction of Belkuchi Upazila in the Sirajganj District, within the Rajshahi Division of Bangladesh. To be eligible, the participants had to be women aged 18 or older who were diagnosed with PCOS based on the Rotterdam criteria, which includes oligo/anovulation, hyperandrogenism, and polycystic ovaries. Additional requirements included having access to a smartphone, the ability to read and write in Bengali, and provided informed consent for their participation in the study.

The exclusion criteria included having a serious medical condition, any previous history of psychiatric illness, participants who took psychiatric medications, participants with extremely severe Dass21 scores, participants who were pregnant, or participants with a history of chronic diseases such as heart, kidney, or respiratory illnesses.

### 2.3. Randomization

A simple randomization technique was performed where a computer-generated random number sequence was used for randomization and numbering. Randomization table preparation, where the study ID was placed along with the assigned intervention to sequentially numbered and sealed envelopes, was performed by an independent statistician who was not involved in the study. Additionally, the statistician made the randomization schedule. These envelopes were matched with the serial number of the PCOS participants and securely stored in an office locker. Group assignments of the control and intervention were concealed in identical and sealed envelopes. The envelops were opened at the time of participant enrollment, which occurred after the patient provided informed verbal consent and was assigned a study ID.

### 2.4. Study activities and content

To measure the stress, anxiety, and depression levels, baseline data collection was performed with the DASS21 questionnaire, which was developed by Lovibond and Lovibond (1995) [27]. The DASS21 questionnaire is a measurement scale for depression, anxiety, and stress, and is comprised of 21 questions. Studies tested DASS21 for reliability and validity with Cronbach's alpha reliability test for both clinical and non-clinical samples. The original version consisted of 42 items (DASS) and the short version consists of 21 items (DASS-21). The Bangla version of DASS21 was validated after using the translated questionnaire among medical students of Bangladesh [28]. DASS21 uses a self-reported scale of three sets of 7 items each, and is designed to measure the depression anxiety and stress. The depression scale evaluates dysphoria, hopelessness, devaluation of life, self-deprecation, lack of interest, anhedonia, and inertia. The anxiety scale measures autonomic arousal, muscle effects, situational anxiety, and anxious affect. The stress scale gauges chronic nonspecific arousal, difficulty relaxing, nervous arousal, and tendencies to be easily upset, agitated, irritable, over-reactive, and impatient [29],[30].

The patient's cortisol level, testosterone hormone level, and BMI were checked at baseline and at endline. The knowledge level of PCOS was evaluated in baseline and at the endline follow up using a validated knowledge questionnaire used in previous studies [20]. The endline data was collected after six months following the intervention.

The questionnaire was translated into Bangla and back-translated to English by the researcher. Then, the developed questionnaire was pretested among diagnosed PCOS participants selected from the gynecology department to check the feasibility, reliability, and validity of the questionnaire as a pilot. This was performed by the researcher and two research assistants. After resolving all issues, the finalized questionnaire was used to collect the data of the randomly recruited participants.

Testosterone (TT), Cortisol, BMI, and waist circumference were measured both in baseline and endline, which is 6 months after the baseline measurement.

The data is collected for both the intervention and control groups.

Two research assistants who were trained by the researcher, collected the data.

From the intervention group, a total of 20 participants were purposively selected to participate in the qualitative component of the study. These participants were chosen from those who completed the full 6-month intervention.

The purpose of the qualitative interviews is to explore barriers and facilitators to using the app and engaging with the intervention along with the contextual factors influencing app usage. An one-on-one; in-depth interview will be conducted with the selected participants via an audio call. All interviews will be audio-recorded using a call recording application, with the participants' consent, to ensure accurate data capture for the subsequent analysis.

Table 1 shows the intervention and control group process for 6 months.

**Table 1.** Study process throughout 6 months.

Schedule	Intervention group	Control group
Baseline sessions	Interview Administered questionnaire Laboratory test (cortisol and testosterone) Smartphone app education Following up phone call from researcher for 6 months	Interview Administered questionnaire Laboratory test (cortisol and testosterone)
Final session	Interview Administered questionnaire Laboratory test (cortisol and testosterone)	Interview Administered questionnaire Laboratory test (cortisol and testosterone)

### 2.5. Training of the research assistants

Two research assistants were responsible for obtaining informed consent, conducting physical examinations, interviewing the participants, installing the app on the phones of those in the intervention group, and guiding them on how to navigate the lessons and understand the PCOS-related health education content. The principal investigator provided comprehensive training for the research assistants, thereby covering both the general and role-specific aspects of the study. General training included the study background, contact details, study structure, and use of technology, while role-specific training focused on recruitment, consent procedures, data collection, electronic data entry in the KOBO toolbox (Kobo toolbox is an app for data collection from fields), storing and having paper forms that can be used as back up, and checking the data and self-evaluation forms for complete data submission. After training, they were evaluated through practice sessions and role-playing in a private setting. The research assistants were both intern doctors at the same hospital and received support from the gynecologist for the participant's referral, selection, and recruitment. Additionally, they were responsible for showing the study participants how to use the app and explaining the contents of the lesson.

### 2.6. Intervention group

A smartphone app (mHealth) that contained 11 lessons, in which two of these featured videos, one presented a storytelling approach to explain PCOS, and the other demonstrated simple home exercises, was installed in the intervention group patients' phones for PCOS related health education. The lesson topics, which focused on PCOS knowledge, are attached as a supplementary file. All app content, including the videos, were delivered in Bengali to ensure accessibility and better understanding for the participants.

The digital health education app contents were completed based on the current evidence and clinical guidelines for PCOS management. Together, the lead researcher, one gynecologist, one nutritionist, two research assistants, supervisors, authors, and co-authors reviewed the contents for cultural appropriateness, medical accuracy, and clarity.

### 2.7. Adherence to intervention

The study maintained monthly follow-ups, during which the lead researcher contacted the participants in the intervention group. This regular engagement supported the participant's adherence to the intervention. Additionally, if a participant missed a scheduled reminder call from the researcher, the research assistants followed up with supplementary calls to ensure continued participation and compliance.

### 2.8. Control group

The control group received usual medical treatments from the gynecologist and were followed up with 6 months after the study period for the endline measurements.

### 2.9. Sample size and power

The sample size was calculated using G* power (version 3.1) for a repeated-measures analysis of variance (ANOVA) with a within-between interaction (F tests). The following parameters were used: two groups, two measurements, non-sphericity correction ε = 1.0, α = 0.05 (Type I error), and power = 0.80 (Type II error = 0.20).

The effect size f = 0.2319 was derived based on prior literature and our study design. Specifically, a previous study reported a Cohen's d of 0.44 [31] for similar outcomes. In our two-group repeated-measures design, the corresponding unadjusted effect size was calculated as follows:

f=d/2=0.44/2=0.22.

We planned to adjust for baseline covariates in the analysis. Assuming that covariates explain 10% of the variance in the outcome (ρ² = 0.10), the adjusted effect size is as follows:

$${\text{f}}_{\text{adj}}=0.22/\surd 1-0.10=0.2319.$$

Using these parameters in G*Power (F tests, repeated-measures ANOVA, within–between interaction), the calculated total sample size required to detect the prespecified effect size with 80% [32] power at α = 0.05 was 148 participants. To account for an expected 20% [33] attrition rate, the sample size was inflated as follows:

$${\text{N}}_{\text{adjusted}}=148/1-0.20=185.$$

Thus, the final recruitment target was 185 participants (93 in the intervention group and 92 in the control group) to ensure sufficient power for the primary analysis.

### 2.10. Blinding

The participants were blinded to the intervention, while the researchers were aware of the group assignments, including enrollment into the intervention and control groups.

The intervention group received the smartphone application and bi-monthly follow-up calls, while the control group continued their usual care. Because the participants did not know whether they belonged to the intervention or control group, blinding at the participant level was maintained.

### 2.11. Outcomes

The primary outcome of this study is the improvement of depression, anxiety, and stress scores, as assessed by the DASS-21 questionnaire.

The secondary outcomes include the following:

- Reduction in serum cortisol and testosterone levels;
- Improved BMI; and
- Enhanced knowledge about PCOS, measured using a structured questionnaire from a previously published study.

## 3. Ethical considerations

Ethical approval was received from the Institutional Review Board of North South University (Approval No. 2024/0RNSU/IRBII 002) and is registered with the Japan Registry of Clinical Trials under the ID jRCT2062240073, https://jrct.mhlw.go.jp/en-latest-detail/jRCT2062240073. The research was conducted in accordance with the principles of the Declaration of Helsinki. Prior to enrollment, all participants were thoroughly informed about the study's purpose, potential risks and benefits, and their rights. Voluntary participation and informed consent were obtained from each participant. Strict confidentiality was maintained for all the study participants throughout the study.

All qualitative and quantitative data was securely stored with restricted access. The participants were informed of their rights, including the voluntary nature of their participation and their ability to withdraw at any time without consequence.

## 4. Quantitative analysis

All data analyses will be performed using the IBM SPSS software (version 26.0, IBM Corp., Armonk, NY, USA). Prior to inferential testing, continuous variables will be evaluated for normality using the Shapiro-Wilk test and inspection of Q-Q plots. When data violate normality assumptions, appropriate transformations (e.g., log or square root) will be applied. If transformation fails to achieve normality, then non-parametric alternatives such as the Wilcoxon signed-rank test (within-group comparisons) and Mann–Whitney U test (between-group comparisons) will be used.

DASS-21 subscales (Depression, Anxiety, Stress) will be separately analyzed as secondary/exploratory using appropriate tests based on normality. A Bonferroni correction will be applied to account for multiple comparisons.

Missing data for primary and secondary outcomes will be handled using multiple imputations, incorporating baseline covariates such as age, BMI, baseline DASS-21 scores, and group assignments to generate plausible values under the assumption of missing at random (MAR). This approach reduces bias and maintains statistical power compared with single-imputation methods such as the last observation carried forward (LOCF). To evaluate the robustness of the findings, sensitivity analyses will be performed under different missing-data assumptions (MCAR and MAR).

All quantitative analyses will be conducted according to the intention-to-treat (ITT) principle, including all randomized participants in their originally assigned groups regardless of adherence to the intervention. The study adopts a two-arm parallel design (intervention and control) with a single primary outcome: participants demonstrating improvement from baseline to the endline score of DASS21. Continuous secondary outcomes, including testosterone and cortisol levels, knowledge scores, and BMI, will be analyzed using a two-way analysis of covariance (ANCOVA) with repeated measures, with the group (intervention and control) as a between-subject factor and time (pre- and post-intervention), and the group × time interaction testing whether the effect of time differs by the group. Potential confounding factors will be adjusted for, and a Bonferroni correction will be applied to account for multiple comparisons. Additionally, exploratory subgroup analyses will be conducted to assess the potential effect modification by age, baseline BMI, and baseline symptom severity. All statistical tests will be two-tailed, with the level of significance set at α = 0.05, unless otherwise specified.

This analytical strategy ensures methodological rigor by addressing statistical assumptions, minimizing false-positive findings, and providing a comprehensive assessment of the digital health education intervention's impact on the mental health and physiological outcomes of women with PCOS.

## 5. Qualitative analysis

Qualitative data will be obtained through semi-structured in-depth interviews conducted post-intervention to explore the participants' experiences, perceptions, and behavioral changes in response to the digital health education platform. The purpose of this component is to gain deeper insight into how women with PCOS engaged with the intervention, perceived its usefulness, and navigated lifestyle and mental health behaviors during and after app usage.

All interviews will be audio-recorded (with participant consent) and transcribed verbatim. Field notes and observational memos will be maintained throughout data collection to capture contextual details. Transcripts will be anonymized by removing all personal identifiers to ensure confidentiality and ethical integrity.

The data will be organized and managed using NVivo or Atlas.ti software. A thematic analysis will be performed following the six-phase framework outlined by Braun and Clarke (2006), which includes familiarization with data, generation of initial codes, theme development, reviewing and refining themes, defining and naming themes, and producing the final report. Two independent researchers will separately code the data, and intercoder reliability will be assessed. Any discrepancies will be discussed until consensus is achieved, thus ensuring analytical reliability and validity.

The coding framework will be iteratively developed, thereby integrating both inductive (data-driven) and deductive (theory-driven) approaches to capture the breadth and depth of the participants' experiences. This process will allow for triangulation between quantitative outcomes and qualitative insights, thereby enhancing the overall interpretation and understanding of the intervention's effectiveness and acceptability.

## 6. Attrition and post randomization bias

Both groups continued to receive routine gynecological care from their attending physicians throughout the study period. The intervention group received additional support through the smartphone-based digital health education program and bi-monthly follow-up calls. The control group only received standard gynecological management but was counselled by research assistants to return for follow-up after 6 months. Although the frequency of physician follow-up in the control group varied, both groups maintained regular contact within the healthcare system to reduce differential attrition.

The frequency of gynecological follow-up in the control group was not standardized and may have varied among participants, which could influence the adherence or outcome assessments. However, efforts were made to reduce attrition bias by counselling all the control group participants to attend a 6-month follow-up and by maintaining an intention-to-treat analysis in data interpretation.

Although an intention-to-treat analysis and data imputation will be employed to minimize attrition-related bias, differences in dropout rates between the intervention and control groups may still introduce post-randomization bias. Future studies could reduce this risk by incorporating balanced engagement strategies for both groups.

## 7. Adverse effect reporting harm

The intervention consists of an app that reports no harm on the study participants. Blood withdrawn for serum cortisol and testosterone are routine procedures for PCOS patients and considered low risk.

## 8. Missing data

Efforts to minimize missing data will include thorough training of the study's personnel, regular data monitoring, and timely follow-ups with the participants to ensure complete and accurate data collection. Missing data for primary and secondary outcomes will be handled using multiple imputation methods, thereby incorporating baseline covariates (e.g., age, BMI, baseline DASS-21 scores, and group assignment) to generate plausible values under the assumption of missing at random (MAR). This approach minimizes bias and preserves statistical power compared with single imputation techniques such as last observation carried forward (LOCF). Sensitivity analyses will be conducted under alternative assumptions of MCAR and MAR to evaluate the robustness of the findings. The DASS-21 will be administered only at baseline and at the endline assessment, and no repeated measures will be collected during the intervention period.

## 9. Discussion

The publication of this research protocol is expected to significantly contribute to the overall impact of the study. This study explores the effect of a mobile application-based health education intervention, combined with researcher-led follow-up, on reducing depression, anxiety, and stress in patients with PCOS. Prior research has demonstrated the effectiveness of mobile health (mHealth) interventions in mitigating mental health issues such as depression, stress, and anxiety. Additionally, evidence supports the positive influence of smartphone-based programs and telephonic counseling in improving the patients' mental health outcomes [34].

This study uniquely integrates mobile app-based education with personalized counseling, thus aiming to evaluate their combined effect on psychological well-being among women with PCOS. Despite increasing awareness, mental health aspects, particularly depression, anxiety, and stress, remain under-discussed, especially in low- and middle-income countries such as Bangladesh [35]. In such contexts, stress and emotional distress are often normalized, thus leaving many individuals unsupported.

Furthermore, awareness of PCOS itself remains limited, even in many developed nations [36]. As a result, opportunities to share knowledge or promote patient education are often missed. Lifestyle modifications tailored to individual needs have been identified as a key approach in effectively managing PCOS [37]. Our study seeks to assess how patients respond to a blended digital and counseling-based intervention to address their mental health challenges.

In Bangladesh, there is no intervention study that has investigated the effects of digital health education on stress, anxiety, and depression among women with PCOS. Our study addresses the research gap by evaluating how a digital education approach influences psychological outcome among Bangladeshi women with PCOS.

Additionally, this study explores the mind-body connection utilizing serum cortisol and testosterone levels as a biological marker measurement together with psychological measures. While Shefin et al. (2024) [38] focused on biochemical parameters, their study lack exploration on how physiological changes relate to emotional or psychological wellbeing.

Current research diverges from previous app development-based interventions guided by the Transtheoretical Model (TTM) [2], which was implemented in other contexts. Our research aims at education-based digital interventions through an app which was tailored to improve awareness, self-efficacy, and mental wellbeing of patients. Thus, this study is a contextually novel relevant approach within Bangladeshi setting where both digital health research and PCOS-related mental health interventions are still emerging.

To the best of our knowledge, this is the first study in Bangladesh to evaluate the impact of a digital health education intervention that specifically targets stress, anxiety, and depression among women with PCOS.

The findings of this study may serve as a valuable reference for clinicians and public health professionals in guiding the future use of digital health education strategies. Specifically, the results can inform decisions regarding the effectiveness, limitations, and advantages of app-based educational interventions, as well as their potential integration into broader healthcare practices. Therefore, this work contributes both contextual innovation and practical insight into scalable, technology-driven mental health support for women with PCOS.

## 10. Results

The baseline data collection was completed in April 2025. Endline data collection began in November 2025. To date, the data analysis has not started. A total of 212 patients were enrolled, with 106 in the control group and 106 in the intervention group.

## 11. Strengths

Using culturally appropriate tools and language is a significant strength of the study. The digital health education intervention was developed in Bangla, the participants' native language, thus ensuring better comprehension even among those with limited digital literacy. A gynecologist and the lead researcher supervised the educational content, while trained research assistants guided the participants in navigating the app and understanding the materials. In addition, the study employed an unbiased sampling method for participant recruitment and used a standardized laboratory to measure the cortisol and testosterone levels, thus enhancing the reliability of the findings.

## 12. Limitations

Despite its strengths, the study has several limitations. The participants were recruited from a single hospital in the Sirajganj district, thus limiting the generalizability of findings to the broader rural PCOS population. Additionally, there was a potential risk of information “spillover” to the control group through patient interactions, although verbal agreements were secured from the participants to minimize this effect. The study explored digital health education delivered via a smartphone app in a rural region of Bangladesh, where most women did not personally own smartphones. This presented a challenge, as low levels of digital literacy slowed the recruitment process. To address this limitation, the researcher conducted monthly follow-up calls to the participants as part of the intervention. Moreover, due to financial and time constraints, the six-month follow-up may not capture the complete long-term effects of the intervention.

Furthermore, since DASS21 is a self-reported measure, it introduces the possibility of response and recall bias, as the participants may underreport or overreport their psychological symptoms based on perceived expectations or social desirability. Although cortisol and testosterone biomarkers partially mitigate this limitation, future studies should consider clinician-rated, ecological momentary assessment tools to strengthen the validity.

Future research should include multi-site randomized controlled trials to improve the generalizability and assess scalability across diverse populations. Incorporating a cost-effectiveness analysis would provide important insights for policymakers and healthcare planners regarding the economic feasibility of implementing digital health education programs for women with PCOS. Moreover, longitudinal designs which assess sustained behavioral and psychological changes beyond the intervention period are warranted.

## 13. Conclusions

The study outcome will help to understand the broader implementation of a dedicated PCOS health app and personalized counseling services to enhance health literacy, encourage proactive health behaviors, and ultimately improve the physical, mental health, and quality of life of PCOS patients.

## Use of AI tools declaration

The authors declare they have not used Artificial Intelligence (AI) tools in the creation of this article.


