# Supplementary material for: Effects of digital health education intervention on stress, anxiety and depression of patients with polycystic ovarian syndrome: study protocol for a single blinded randomized controlled trial
Source: AIMS Public Health. 2026 Jan 9;13(1):105–20. doi: 10.3934/publichealth.2026007 (PMC13084743; doi:10.3934/publichealth.2026007)
Supplement: Supplementary file 1 [file publichealth-13-01-007-s001.pdf]

Public Health

AIMS Public Health, 13(1): 105–120.
DOI: 10.3934/publichealth.2026007
Received: 17 August 2025
Revised: 31 October 2025
Accepted: 13 November 2025
Published: 09 January 2026

https://www.aimspress.com/journal/aimsph

Protocol

Effects of digital health education intervention on stress, anxiety and depression of patients with polycystic ovarian syndrome: study protocol for a single blinded randomized controlled trial

Sadia Akter[1], Farkhanda Mahjebin[2], Mohammad Delwer Hossain Hawlader[3,4], Md Moshiur Rahman[5], Sanmei Chen[5], Saori Kashima[6,7], Sheuly Akter[2], Shamit Dasgupta[8] and Yoko Shimpuku[1,5,*]

[1] Graduate School of Innovation and Practice for Smart Society, Hiroshima University, Japan
[2] Khwaja Yunus Ali Medical College and Hospital, Bangladesh
[3] Department of Public Health, North South University, Bangladesh
[4] NSU Global Health Institute (NGHI), North South University, Dhaka 1229, Bangladesh
[5] Graduate School of Biomedical and Health Sciences, Hiroshima University, Japan
[6] Center for the Planetary Health and Innovation Science, The IDEC Institute, Hiroshima University, Japan
[7] Environmental Health Sciences Laboratory, Graduate School of Advanced Science and Engineering, Hiroshima University, Japan
[8] Ministry of Health, Government of British Columbia, Canada

* **Correspondence:** Email: yokoshim@hiroshima-u.ac.jp; Tel: +3232759774; Fax: +3232759774.

Supplementary

Table 1. SPIRIT 2025 checklist of items to address in a randomized trial protocol*.

Section / Topic	No	SPIRIT 2025 checklist item description	Reported on page no.
Administrative information			

Title and structured summary	1a	Title stating the trial design, population, and interventions, with identification as a protocol	1,2,3
	1b	Structured summary of trial design and methods, including items from the World Health Organization Trial Registration Data Set	2,3
Protocol version	2	Version date and identifier	3
Roles and responsibilities	3a	Names, affiliations, and roles of protocol contributors	1,18
	3b	Name and contact information for the trial sponsor	1,17,18
	3c	Role of trial sponsor and funders in design, conduct, analysis, and reporting of trial; including any authority over these activities	18
	3d	Composition, roles, and responsibilities of the coordinating site, steering committee, endpoint adjudication committee, data management team, and other individuals or groups overseeing the trial, if applicable	18
Open science			
Trial registration	4	Name of trial registry, identifying number (with URL), and date of registration. If not yet registered, name of intended registry	11
Protocol and statistical analysis plan	5	Where the trial protocol and statistical analysis plan can be accessed	10,11
Data sharing	6	Where and how the individual de-identified participant data (including data dictionary), statistical code, and any other materials will be accessible	10,11
Funding and conflicts of interest	7a	Sources of funding and other support (e.g., supply of drugs)	18
	7b	Financial and other conflicts of interest for principal investigators and steering committee members	18
Dissemination policy	8	Plans to communicate trial results to participants, healthcare professionals, the public, and other relevant groups (e.g., reporting in trial registry, plain language summary, publication)	14
Introduction			
Background and rationale	9a	Scientific background and rationale, including summary of relevant studies (published and unpublished) examining benefits and harms for each intervention	2
	9b	Explanation for choice of comparator	6,9,10
Objectives	10	Specific objectives related to benefits and harms	11
Methods: Patient and public involvement, trial design			
Patient and public involvement	11	Details of, or plans for, patient or public involvement in the design, conduct, and reporting of the trial	16
Trial design	12	Description of trial design including type of trial (e.g., parallel group, crossover), allocation ratio, and framework (e.g., superiority, equivalence, non-inferiority, exploratory)	7,8
Methods: Participants, interventions, and outcomes			
Trial setting	13	Settings (e.g., community, hospital) and locations (e.g., countries, sites) where the trial will be conducted	6
Eligibility criteria	14a	Eligibility criteria for participants	7,8
	14b	If applicable, eligibility criteria for sites and for individuals who will	6,8,9,10

		deliver the interventions (e.g., surgeons, physiotherapists)	
Intervention and comparator	15a	Intervention and comparator with sufficient details to allow replication including how, when, and by whom they will be administered. If relevant, where additional materials describing the intervention and comparator (e.g., intervention manual) can be accessed	9,10
	15b	Criteria for discontinuing or modifying allocated intervention/comparator for a trial participant (e.g., drug dose change in response to harms, participant request, or improving/worsening disease)	6,7
	15c	Strategies to improve adherence to intervention/comparator protocols, if applicable, and any procedures for monitoring adherence (e.g., drug tablet return, sessions attended)	8,9
	15d	Concomitant care that is permitted or prohibited during the trial	10
Outcomes	16	Primary and secondary outcomes, including the specific measurement variable (e.g., systolic blood pressure), analysis metric (e.g., change from baseline, final value, time to event), method of aggregation (e.g., median, proportion), and time point for each outcome	10
Harms	17	How harms are defined and will be assessed (e.g., systematically, non-systematically)	13
Participant timeline	18	Time schedule of enrollment, interventions (including any run-ins and washouts), assessments, and visits for participants. A schematic diagram is highly recommended (see Figure)	5,8,9
Sample size	19	How sample size was determined, including all assumptions supporting the sample size calculation	8,9,10
Recruitment	20	Strategies for achieving adequate participant enrollment to reach target sample size	8
Methods: Assignment of interventions			
Randomization:			
Sequence generation	21a	Who will generate the random allocation sequence and the method used	6,7,8
	21b	Type of randomization (simple or restricted) and details of any factors for stratification. To reduce predictability of a random sequence, other details of any planned restriction (e.g., blocking) should be provided in a separate document that is unavailable to those who enroll participants or assign interventions	6,7,8
Allocation concealment mechanism	22	Mechanism used to implement the random allocation sequence (e.g., central computer/telephone; sequentially numbered, opaque, sealed containers), describing any steps to conceal the sequence until interventions are assigned	6,7,8
Implementation	23	Whether the personnel who will enroll and those who will assign participants to the interventions will have access to the random allocation sequence	7,8
Blinding	24a	Who will be blinded after assignment to interventions (e.g., participants, care providers, outcome assessors, data analysts)	10
	24b	If blinded, how blinding will be achieved and description of the similarity of interventions	10

	24c	If blinded, circumstances under which unblinding is permissible, and procedure for revealing a participant's allocated intervention during the trial	10
Methods: Data collection, management, and analysis			
Data collection methods	25a	Plans for assessment and collection of trial data, including any related processes to promote data quality (e.g., duplicate measurements, training of assessors) and a description of trial instruments (e.g., questionnaires, laboratory tests) along with their reliability and validity, if known. Reference to where data collection forms can be accessed, if not in the protocol	6,7,8,9
	25b	Plans to promote participant retention and complete follow-up, including list of any outcome data to be collected for participants who discontinue or deviate from intervention protocols	9
Data management	26	Plans for data entry, coding, security, and storage, including any related processes to promote data quality (e.g., double data entry; range checks for data values). Reference to where details of data management procedures can be accessed, if not in the protocol	12
Statistical methods	27a	Statistical methods used to compare groups for primary and secondary outcomes, including harms	10,11,12
	27b	Definition of who will be included in each analysis (e.g., all randomized participants), and in which group	10,11,12
	27c	How missing data will be handled in the analysis	11,12,14
	27d	Methods for any additional analyses (e.g., subgroup and sensitivity analyses)	11,12
Methods: Monitoring			
Data monitoring committee	28a	Composition of data monitoring committee (DMC); summary of its role and reporting structure; statement of whether it is independent from the sponsor and funder; conflicts of interest and reference to where further details about its charter can be found, if not in the protocol. Alternatively, an explanation of why a DMC is not needed	18
	28b	Explanation of any interim analyses and stopping guidelines, including who will have access to these interim results and make the final decision to terminate the trial	12
Trial monitoring	29	Frequency and procedures for monitoring trial conduct. If there is no monitoring, give explanation	18
Ethics			
Research ethics approval	30	Plans for seeking research ethics committee/institutional review board approval	11
Protocol amendments	31	Plans for communicating important protocol modifications to relevant parties	13,18
Consent or assent	32a	Who will obtain informed consent or assent from potential trial participants or authorized proxies, and how	7,8,9
	32b	Additional consent provisions for collection and use of participant data and biological specimens in ancillary studies, if applicable	7,8,9

Confidentiality	33	How personal information about potential and enrolled participants will be collected, shared, and maintained in order to protect confidentiality before, during, and after the trial	10
Ancillary and post-trial care	34	Provisions, if any, for ancillary and post-trial care, and for compensation to those who suffer harm from trial participation	18

Storytelling app

The app containing story telling video that is easily understandable for the general population and is in Bengali language. An education platform application for smartphones is being used for this study. The previously developed educational platform app named Goocus is being used. Along with the storytelling app there is a home exercise video showing simple exercise one can do just being at home. The lesson contents provide PCOS related information and simple daily lifestyle advice that can be easily achievable for women (Figure 1).

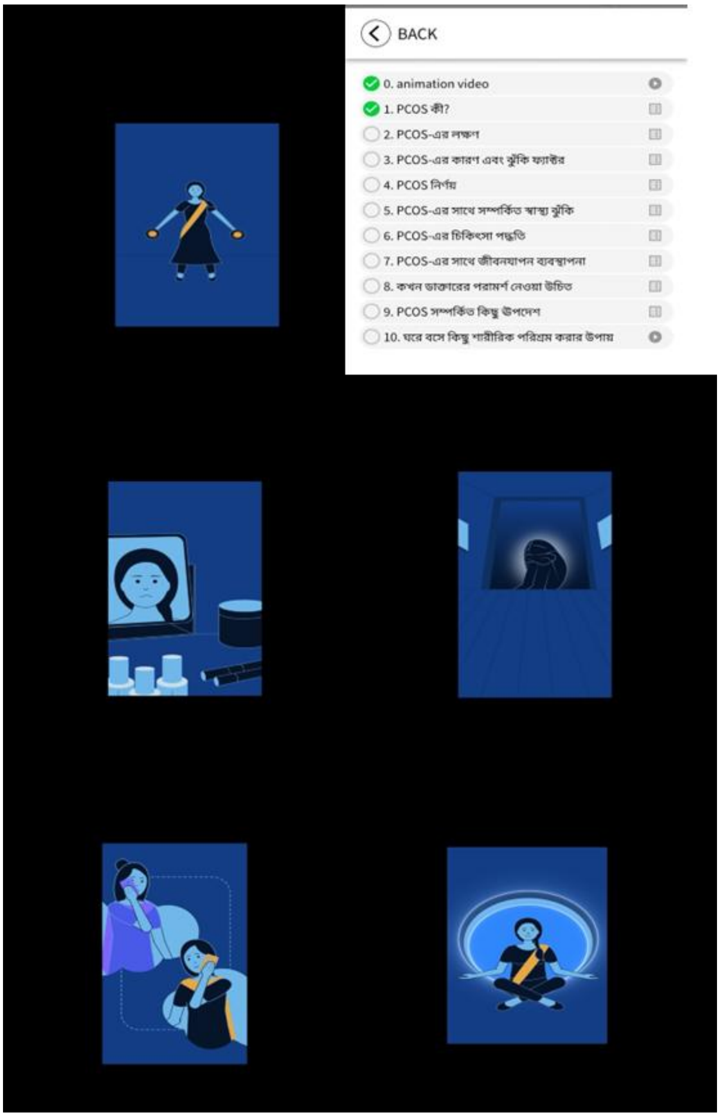

Figure 1. Pictures showing the app content.

Lesson content

The contents of the lesson are listed below in Table 2.

Table 2. Contents of the app.

No.	Contents
Lesson 1	Animation video
Lesson 2	What is PCOS
Lesson 3	Symptoms of PCOS
Lesson 4	Cause and risk factors of PCOS
Lesson 5	Diagnosis of PCOS
Lesson 6	Health risks associated with PCOS
Lesson 7	Treatment of PCOS
Lesson 8	Managing PCOS with lifestyle changes
Lesson 9	When to see a doctor
Lesson 10	Lifestyle advice/suggestion
Lesson 11	Home exercise video

1. What is PCOS?

PCOS is a common hormonal disorder affecting women of reproductive age. It is characterized by the presence of multiple small cysts on the ovaries, irregular menstrual cycles, excess levels of male hormones (androgens), and insulin resistance. These factors can lead to a variety of symptoms and complications over time.

2. Symptoms of PCOS

- Irregular periods: Women with PCOS may experience fewer periods (oligomenorrhea) or no periods at all (amenorrhea).
- Excess androgen: Elevated levels of male hormones can lead to physical signs such as excessive hair growth (hirsutism), acne, and male-pattern baldness.
- Polycystic ovaries: Enlarged ovaries with multiple small cysts may be detected via ultrasound.
- Weight gain: Many women with PCOS struggle with weight gain, especially around the abdomen.
- Infertility: Due to irregular ovulation, many women with PCOS may have difficulty conceiving.
- Mental health issues: Women with PCOS are at an increased risk of experiencing depression, anxiety, and mood disorders. Studies suggest that the hormonal imbalances, body image concerns due to weight gain or hirsutism, and fertility issues may contribute to psychological distress in women with PCOS [1].

3. Causes and risk factors: The exact cause of PCOS is unclear, but several factors play a role

- Hormonal imbalance: Higher-than-normal androgen levels interfere with the ovaries' ability to function properly.

- Insulin resistance: Many women with PCOS are insulin resistant, meaning their bodies can't use insulin effectively. This leads to higher blood sugar levels, which in turn causes the body to produce more insulin, further driving up androgen production.
- Genetics: PCOS tends to run in families, suggesting a genetic link.

4. Diagnosis PCOS is typically diagnosed through

- Medical history and symptoms: A doctor will inquire about menstrual cycles, weight changes, symptoms of excess androgens, and psychological health.
- Physical exam: Including checking for signs of excess hair growth, acne, weight gain, and mood disturbances.
- Blood tests: To measure hormone levels, glucose, cholesterol, and potential indicators of mental health conditions like depression or anxiety.
- Pelvic ultrasound: To examine the ovaries for cysts and assess their size.

5. Health risks associated with PCOS: PCOS is linked to several long-term health conditions

- Type 2 diabetes: Due to insulin resistance.
- Cardiovascular disease: Increased risk due to factors like obesity, high blood pressure, and high cholesterol.
- Endometrial cancer: Irregular menstruation can increase the risk of uterine lining buildup (endometrial hyperplasia), which may lead to cancer.
- Sleep apnea: Especially in overweight women with PCOS.
- Mental health disorders: Higher incidences of depression, anxiety, and eating disorders are common in women with PCOS. Hormonal imbalances, metabolic issues, and concerns about body image contribute to this increased mental health burden [2].

6. Treatment options while there is no cure for PCOS, treatments can help manage symptoms

- Lifestyle changes: Weight loss through diet and exercise can significantly improve symptoms and regulate menstrual cycles.
- Medications: (All medications should be certified doctor suggested)
  - Birth control pills to regulate periods and reduce androgen levels.
  - Metformin, which helps improve insulin sensitivity.
  - Anti-androgens to reduce hirsutism and acne.
  - Antidepressants or anxiolytics may be prescribed for women dealing with depression or anxiety linked to PCOS [3].
  - Fertility treatments like clomiphene or in vitro fertilization (IVF) for women trying to conceive.
- Therapy: Cognitive-behavioral therapy (CBT) and counseling can be effective in managing anxiety, depression, and body image concerns associated with PCOS [3].
- Surgical options: In some cases, a procedure called laparoscopic ovarian drilling (LOD) may be considered to restore ovulation.

7. Managing PCOS with lifestyle changes

- Diet: A balanced diet focusing on whole grains, lean proteins, and plenty of vegetables can help manage weight and improve insulin sensitivity.
- Exercise: Regular physical activity can help with weight loss, reduce insulin resistance, and improve mental well-being by reducing stress and anxiety.
- Mental health support: Women with PCOS are at a higher risk of depression and anxiety, so seeking counseling or support is important. Mindfulness practices, meditation, and yoga may also help alleviate stress and improve emotional health [4].

8. When to see a doctor

If you experience irregular periods, difficulty conceiving, unexplained weight gain, signs of excess androgens like hirsutism or severe acne, or mental health concerns like depression or anxiety, it is essential to consult a healthcare professional.

Lifestyle guideline to manage PCOS symptoms

1. Drink at least 8 glasses of water daily.
2. Engage in 30 minutes of exercise.
3. Follow a diet low in carbohydrates and high in protein.
4. Eat fresh fruits and vegetables: vitamin C sources like oranges and lemons, magnesium-rich foods such as spinach, bananas, and pineapples, and chromium sources like garlic and onions.
5. Limit your intake of coffee and cola.
6. opt for natural herbal tea instead.
7. Reduce smoking and alcohol consumption.
8. Practice breathing exercises twice a day.
9. Use a video for breathing exercises.
10. Aim for 7–8 hours of sleep at night.
11. Avoid screens before bedtime.
12. Refrain from staying up late
13. Reduce your intake of processed foods and focus on consuming more naturally grown foods.
14. Practice Self-counseling.
15. Ten thousand steps per day or any type of exercise, such as swimming, yoga, gym, anything you like to do.
16. Don't take exercise as stress or must to do, do it as you love to do it. If anything, that makes you stressed and not calm, stop doing it immediately. Stress causes increase cortisol which affects your health.
17. Limit oily food. Limit processed food, eat more nature grown food.
18. Limit sitting time, lying time.
19. Seek help from a certified psychologist or counselor. Practice positive self-talk, self-affirmation, self-assurance. talk to certified nutritionist, gynecologist, endocrinologist.
20. You know your body better than anyone else. Try to help and take care of your body and mind.
21. Try to have yogurt once a day.

22. Peanuts, fruits are good source of energy rather than burgers and pizzas.
23. It is difficult to lose or gain weight who have polycystic ovarian syndrome. Take baby step. Start today. Be consistent. Don't lose hope.

Source of lesson content

1. Dokras A, Clifton S, Futterweit W, et al. (2012) Increased prevalence of anxiety symptoms in women with polycystic ovary syndrome: Systematic review and meta-analysis. *Fertil Steril* 97: 225–30.e2. https://doi.org/10.1016/j.fertnstert.2011.10.022
2. Månsson M, Holte J, Landin-Wilhelmsen K, et al. (2008) Women with polycystic ovary syndrome are often depressed or anxious, A case control study. *Psychoneuroendocrinology* 33: 1132–1138. https://doi.org/10.1016/j.psyneuen.2008.06.003
3. Dokras A (2012) Mood and anxiety disorders in women with PCOS. *Steroids* 77: 338–341. https://doi.org/10.1016/j.steroids.2011.12.008
4. Young CC, Monge M, Minami H, et al. (2022) Outcomes of a Mindfulness-Based Healthy Lifestyle Intervention for Adolescents and Young Adults with Polycystic Ovary Syndrome. *J Pediatr Adolesc Gynecol* 35: 305–313. https://doi.org/10.1016/j.jpag.2021.10.016

AIMS Press

© 2026 the Author(s), licensee AIMS Press. This is an open access article distributed under the terms of the Creative Commons Attribution License (http://creativecommons.org/licenses/by/4.0)
